# Recent advancement in inflammatory biomarkers for cervical cancer diagnosis and therapy

**DOI:** 10.3389/fonc.2026.1806448

**Published:** 2026-08-13

**Authors:** Utkarsha Sahu, Sumeet Singh, Surbhi Jain, Mukesh Samant, Prashant Khare

**Affiliations:** 1 Department of Microbiology, All India Institute of Medical Sciences, Bhopal, Madhya, India; 2 Xenesis Institute, Absolute, Gurugram, Haryana, India; 3 Cell and Molecular Biology Laboratory, Department of Zoology, Soban Singh Jeena University, Almora, Uttarakhand, India; 4 Department of Zoology, DSB Campus, Kumaun University, Nainital, Uttarakhand, India

**Keywords:** biomarkers, cervical cancer, diagnosis, inflammation, therapy

## Abstract

**Background:**

Cervical cancer (CC) ranks as the fourth most prevalent malignancy among women. High-risk human papillomavirus (HPV) infection is a significant contributor to CC in economically disadvantaged nations. The incidence of CC may be significantly reduced by preventive measures, and early detection is strongly associated with improved survival rates. CC presents a substantial danger to women in low- and middle-income countries who often have severe and untreatable sicknesses due to limited resources.

**Results:**

Inflammatory markers, such as platelet count, platelet-to-lymphocyte ratio, and IL-6 tumoral expression, indicate a worse likelihood of survival in individuals with CC. Single nucleotide polymorphisms in cytokine genes have been linked to an increased risk of CC. Cytokines such as IL-12, IL-6, and IL-10 may serve as biomarkers for CC. The prognosis of CC may also be influenced by DNA methylation. Immune cells possess a unique ability to combat tumors, and their interactions with tumor growth and invasion significantly impact prognosis and treatment. Genetic biomarkers, such as circulating cell-free or cellular DNA, RNA, SNPs, and epigenetic biomarkers like microRNA and DNA methylation, have the potential to forecast the likelihood of developing a disease. Protein and cell biomarkers, such as cytokines, hormones, and enzymes, have the potential to detect cancer at an early stage and provide accurate diagnosis and prognosis.

**Conclusion:**

This review focuses on evaluating several inflammatory biomarkers with potential use in diagnosing and targeting specific therapeutic interventions for the successful treatment of CC, highlighting the attractive prospects of their medicinal utilization.

## Highlights

- A variety of biomarkers are being discovered and evaluated for risk prediction, diagnosis, prognosis, and response prediction.
- Genetic biomarkers, such as circulating cell-free/cellular DNA, RNA, SNPs, and epigenetic biomarkers, such as miRNAs and DNA methylation, are promising risk-prediction biomarkers.
- Protein and cell biomarkers, such as cytokines, antibodies, hormones, and enzymes, can predict accurate and timely cancer diagnosis and prognosis efficiently. These biomarkers can also be used as therapeutic targets for treating CC.
- Despite extensive research being conducted on cancer biomarkers and biosensors, further pre-clinical research, clinical trials, and population-based studies are warranted to develop population screening methods for risk prediction and early diagnosis of CC.

## 1 Introduction

Cervical cancer (CC) is the fourth most common malignancy in women, with an estimated 662,044 cases and 348,709 deaths from cervical cancer occurring in 2022, according to GLOBOCAN 2022 (1). CC is also the second most common malignancy in female in reproductive age. By the year 2050, the number of CC cases and fatalities is expected to rise by 56.8% and 80.7%, respectively (1). According to the latest WHO data, CC remains concentrated in low- and middle-income countries, which bear the highest incidence and mortality. According to GLOBOCAN 2022 data, the incidence of CC in India reached 127,526 cases, with a corresponding mortality of 79,906 deaths, making it one of the key burdens on the health system (2). High-risk human papillomavirus (HR-HPV) infection has been identified as a key factor in the development of CC (3). Furthermore, genetic and epigenetic changes in host cell genes play a role in the progression of cervical precancerous lesions to invasive carcinoma. Most HPV infections resolve without any clinical signs or symptoms within 6–18 months of acquisition (transient infections). However, some infections persist and increase the likelihood of premalignant or malignant disease (4).

**Figure 1:**
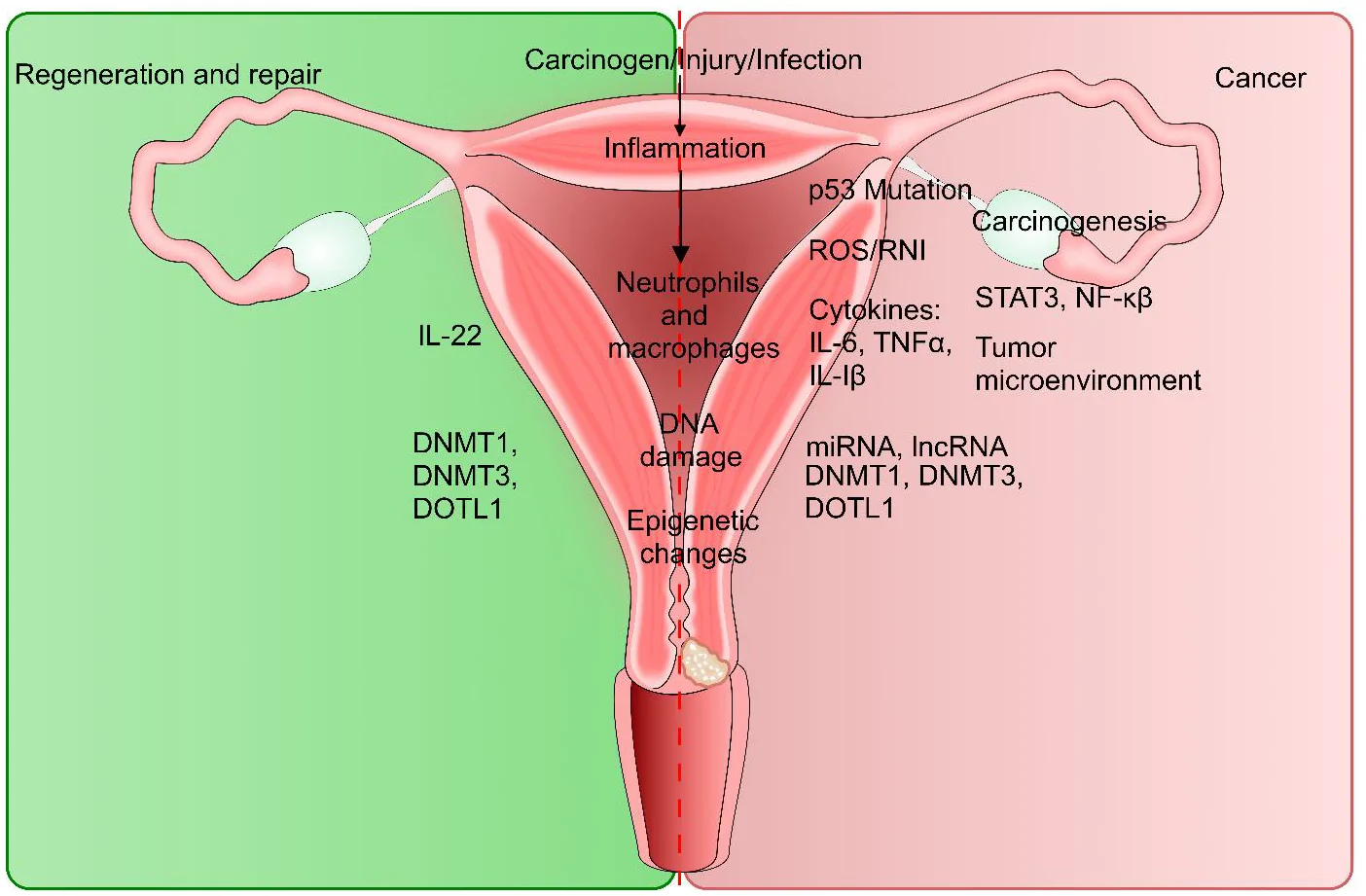
Comparison of tissue damage response in cervical cancer during regeneration and repair versus cervical cancer. In response to external stress, including carcinogens, injury, and HPV infection, tissue generates inflammation, which further triggers an immune response and can lead to DNA damage and epigenetic changes. The key immune cells activated in response to external stress are neutrophils and macrophages. Gene expression alterations are facilitated by important epigenetic regulators such as DNMT1, DNMT3, and DOT1, and tissue regeneration and repair are promoted by the cytokine IL-22. p53 mutation, increase in ROS/RNI, and various cytokines such as IL-6, TNF-α, and IL-1β further contribute to uncontrolled cell division, promoting the initiation and formation of tumors.

Squamous cells respond to external stressors (carcinogens, injury, or HPV infection) by activating immune cells, repairing DNA damage, undergoing epigenetic modifications, and facilitating tissue repair and survival (**Figure 1**). Excessive survival signaling may lead to CC progression. The progression of CC is a multistep process that includes infection with oncogenic HPV, the production of cervical intraepithelial lesions, *in situ* carcinoma/neoplastic growth, and invasion. (5). Microwounds allow HPV to infiltrate basal epithelial cells. The virus then uses nuclear envelope breaks to integrate its genome into the host’s genome in the nucleus. HPVs take control of the host genome once they enter the nucleus, self-replicate, and spread throughout the epithelium (6). In comparison to those of normal cells, host cells develop unevenly and in a disorderly manner as the viral genome replicates. The virions are then sloughed off with the host epithelium’s dead squamous cells, allowing for continued transmission. The oncoproteins E6 and E7 disrupt cell cycle checkpoint control by inactivating p53 and pRb and blocking CDK inhibitors, such as p16, p21, and p27, allowing cells to grow and proliferate more freely. As a result, E6 and E7 alter the integrity of the cell cycle and contribute to cellular immortalization and transformation.

Cervical carcinogenesis progresses through a series of molecular processes. During CC progression, increased expression of genes associated with cell proliferation, angiogenesis, DNA repair, cell cycle progression, mitogenesis, and growth factor activity has been shown in various studies (7, 8). It is evident that innate immunity, inflammation, and cancer are directly related; however, the exact molecular and cellular mechanisms involved are still elusive (9) (10). Inflammatory biomarkers, such as a higher platelet count and Platelet-to-Lymphocyte Ratio (PLR) before treatment, as well as higher IL-6 tumoral expression, predict poor overall survival in patients with CC (11). Here, we review a variety of inflammatory biomarkers for CC diagnosis and therapy.

Several recent articles have explored the landscape of CC biomarkers from different perspectives, such as immune cell dynamics, clinical translation and circulating inflammatory markers (12, 13). The current review article expands upon those articles specifically by outlining inflammatory biomarkers, with clinically relevant framework related with tumor immune microenvironment, cytokine response, HPV-triggered inflammatory pathways, hematological indices and immunotherapy response markers.

## 2 Biomarkers associated with cervical cancer

**Figure 2:**
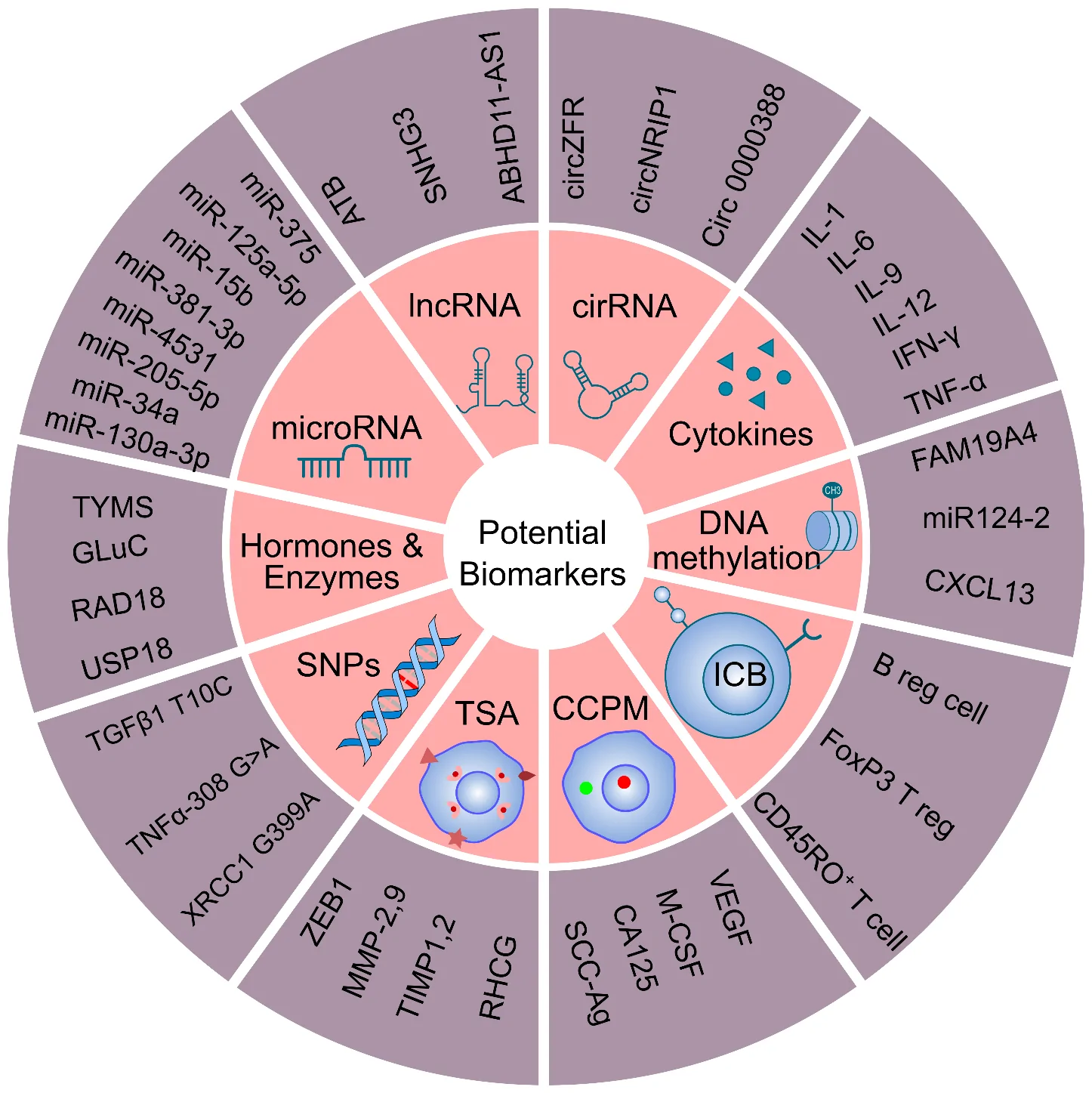
Potential biomarkers for cervical cancer. ICB, Immune cell biomarker; CCPM, Cancer cell proliferation marker; TSA, Tumor specific antigen.

Numerous biomarkers associated with CC have been identified and are essential for understanding CC progression, prognosis, and therapeutic response. In this review, we have discussed in length about various biomarkers associated with CC, including Single Nucleotide Polymorphisms (SNPs), DNA methylation, immune cell biomarkers, cytokines, non-coding RNAs, hormones, enzymes, tumor-specific antigens, and cancer cell proliferation markers (**Table 1**; **Figure 2**). These biomarkers can help in understanding the molecular landscape of CC and can also help in developing new therapeutic targets for personalized medicine.

**Table 1:** Potential biomarker in cervical cancer.

Category	Potential biomarker	Sample type	Detection method	Study phase	Clinical application	Function/significance	Risk association	Reference
SNPs	TGFB1 T10C, XRCC1 G399A	Genomic DNA (Peripheral blood)	Direct sequencing (Sanger)	Phase I, single-center case-control (n=545, Saudi cohort)	Risk stratification; population-level susceptibility marker	TGFB1 T10 (Leu) and XRCC1 399A (Gln) alleles elevate the risk of cervical cancer by 1.5-fold.	Increased Risk	(14)
	TNF-308α G>A	Genomic DNA	TaqMan real-time PCR	Phase I, case-control (91 CC vs. 161 controls)	Risk stratification; candidate pharmacogenomic marker	linked to the risk of cervical cancer	Increased Risk	(15)
	IL-10 (rs1800896)	Genomic DNA (cervical cells)	TaqMan real-time PCR	Phase I case-control (n=315)	Risk stratification; cytokine pathway biomarker	Associated with increased inflammatory cytokines and heightened risk of cervical cancer.	Increased Risk	(16)
	IL-6 (rs1800795, rs1800796, rs1800797)	Genomic DNA (pooled GWAS meta-dataset)	GWAS genotyping arrays (meta-analysis of 118 studies)	Phase II large-scale meta-analysis (50,053 cases/65,204 controls)	Population-level cancer risk stratification	Linked to an elevated cancer risk and can serve as predictive biomarkers.	Increased Risk	(17)
DNA methylation	FAM19A4 and miR124–2 gene methylation	Cervical scrape (archived, HPV-positive screening cohort, POBASCAM trial)	Methylation-specific PCR/bisulfite sequencing	Phase II/III longitudinal RCT cohort (POBASCAM, n=1,040; 14-yr follow-up)	Triage in HPV+ screening; rules out CIN3+/CC	FAM19A4 methylation can be used for secondary cervical (pre)cancer screening.	Decreased risk/favorable (negative result)	(18, 19)
	CXCL13 hypermethylation	CC, CIN, and normal cervical epithelial (NCE) tissues; CC cell lines; xenograft model	IHC; flow cytometry; methylation-specific PCR; functional assays	Phase I case-series with *in vitro*/*in vivo* functional validation; TCGA analysis	Prognostic marker; potential immunotherapy response predictor	CXCL13 downregulation was linked to hypermethylation in cervical cancer.	Increased risk (promoter silencing)	(20)
Immune cell	regulatory B cells	Peripheral blood	flow cytometry for Breg cells	Phase I multiple independent case-control studies	Immune monitoring; prognostic stratification; therapeutic target (blockade)	Associated with the enhancement of metastasis through the reduction of perforin- and granzyme B-secreting CD8+ T cells	Increased risk/poor prognosis	(21)
Cytokine	ratios of CD4+CD25+ regulatory T cells in the peripheral blood and the serum IL-10 concentration	Peripheral blood	Flow cytometry; ELISA	Phase I case-control with survival data (n=70 CC vs. 70 controls)	Prognostic biomarker; treatment response monitoring	Reduced IL-10 expression correlates with an increased likelihood of survival.	Favorable prognosis (low IL-10)	(22)
	reduced IL-1β and IL-18 expression	Cervical tissue biopsy	qPCR (mRNA expression)	Phase I case-series (n=74; normal, LSIL, HSIL, cancer groups)	Progression risk stratification (pre-neoplastic → invasive)	The likelihood of preneoplastic lesions advancing to cancer was 2.5 and 2.08 times greater, respectively.	Increased risk (deficient early response)	(23)
miRNA	miR-34a	Cervical exfoliated cells (qRT-PCR)	qRT-PCR	Phase I case-control, HPV-genotype stratified (HPV16/52/58)	HPV genotype-specific risk stratification; CC vs. CIN distinction	May be associated with the initiation and advancement of cervical cancer	Increased risk (downregulated)	(24)
	miR-205-5p, miR-130a-3p, miR-4531, and miR-381-3p	Cervical sample (liquid-based cytology)	NanoString nCounter miRNA Expression Assay	Phase I case-control (CIN3 n=20 vs. HFS n=11)	CIN3 detection; molecular triage in LBC samples	May serve as biomarkers for CIN 3	Increased risk	(25, 26)
	miR-155	Cervical tissue and peripheral blood	qRT-PCR (blood/Pap smear); qPCR (FFPE); Western blot; flow cytometry	Phase I/II (3 independent datasets) across 3 sample types	Early detection (LSIL stage); liquid biopsy candidate; mechanistic therapeutic target (SOCS1/STAT3)	overexpressed in peripheral blood, reduces SOCS1 expression and facilitate a Th17/Treg imbalance	Increased risk/poor prognosis	(27, 28)
	miR-34a-5p and miR-218-5p	Paired urine, serum, cervical scrape, tumor tissue (qRT-PCR)	qRT-PCR	Phase I/II multi-sample type concordance study	Non-invasive detection (urine); prognostic stratification	Non-invasive urine samples may facilitate the reliable identification of these biomarkers for the early diagnosis and prognosis of cervical cancer, serving as an independent predictor of overall survival in patients.	Favorable prognosis (downregulation = worse)	(29)
	miR-92a-5p and miR-155-5p	Pap smear	Real-time PCR (qRT-PCR	Phase I case-control (LSIL n=25, HSIL n=25, controls n=25)	Early detection of CIN; triage from normal cytology	Elevations in HPV-associated low-grade squamous intraepithelial lesions (LSILs) and high-grade squamous intraepithelial lesions (HSILs)	Increased risk	(30)
	hsa-miR-26b-5p, hsa-miR-146b-5p, hsa-miR-191-5p, hsa-miR-484, hsa-miR-574-3p, and hsa-miR-625-3p	Blood-based liquid biopsy miRNAs extracted from plasma samples	RT-qPCR; random forest classifier	Phase II three-cohort design (screening n=24, validation n=380, independent validation n=47)	Non-invasive CC/CIN screening classifier	Associated with cervical lesions and may serve as non-invasive biomarkers.	Increased risk	(31)
lncRNA	SNHG3	CC tumor tissue	qRT-PCR; Western blot; *in vitro*/*in vivo* functional assays	Phase I mechanistic case-series with functional validation	Prognostic biomarker; therapeutic target (YAP1/Hippo axis)	Small Nucleolar RNA Host Gene 3 (SNHG3) inhibits YAP1 and also affects the transcription of other YAP1 target genes.	Increased risk (oncogenic)	(32)
	ABHD11 antisense RNA 1	CC tumor tissue and cell lines				Competitively sequestering miR-330-5p to enhance MARK2 expression, indicating that ABHD11-AS1 may serve as a biomarker for colorectal cancer.	Increased risk (oncogenic)	(33)
	SPINT1-AS1	Cervical cancer tissue; CC cell lines (*in vitro*); xenograft model (*in vivo*)	qRT-PCR; RIP assay; luciferase; *in vitro*/*in vivo* assays	Phase I mechanistic case-series with functional validation	Prognostic biomarker; therapeutic target (miR-214/Wnt-β-catenin axis)	Suppresses miR-214 synthesis while enhancing Wnt signaling, suggesting its potential as a predictive biomarker and therapeutic target for cervical cancer	Increased risk (oncogenic)	(34)
circRNA	hsa circ 0025721	Cervical cancer tissue (RNA-seq + bioinformatics)	RNA-seq; bioinformatic ceRNA network modelling	Phase I bioinformatic	Exploratory TME immune risk modelling; immunotherapy predictor	May upregulate CXCL8 by sponging miRNAs such as hsa-miR-4428	Increased risk	(35)
	circNRIP1	Cervical cancer tissue; CC cell lines (SiHa vs. H8; qRT-PCR); *in vitro* and *in vivo* models	circRNA microarray; qRT-PCR; functional assays (migration, invasion, proliferation); *in vivo* xenograft	Phase I mechanistic case-series with functional validation	Prognostic biomarker (lymphovascular invasion); therapeutic target	Modulates the PTP4A1/ERK1/2 pathway, at least partially, by sequestering miR-629-3p. May serve as a prognostic biomarker and therapeutic target for patients with cervical cancer	Increased risk	(36).
	Circ 0000388	CC clinical tissue samples; CC cell lines	qRT-PCR; CCK-8; TUNEL; wound healing; transwell; RIP; luciferase	Phase I mechanistic case-series with *in vitro* functional validation	Exploratory diagnostic and therapeutic target	facilitates cancer progression through the regulation of miR-337-3p and TCF12	Increased risk	(37)
	circZFR	Cervical cancer tissue	circRNA microarray; qRT-PCR; circRNA pull-down; mass spectrometry; circRIP; Co-IP; *in vitro*/*in vivo* assays	Phase I/II two independent cohorts + GEO validation + functional validation	Prognostic biomarker; therapeutic target (cell cycle); candidate liquid biopsy marker	Interacts with SSBP1 and facilitates the assembly of CDK2/cyclin E1 complexes.	Increased risk (oncogenic)	(38)
Hormones and Enzymes	Thymidylate Synthetase (TYMS)	Multi-dataset bioinformatic analysis; CC cell lines; clinical tissue data	qRT-PCR, functional knockdown/overexpression	Phase I bioinformatic discovery + *in vitro* functional validation	Prognostic biomarker; potential therapeutic target (chemosensitivity)	miR-197-3p/TYMS axis regulates the degradation of cervical cancer cells, establishing a foundation for molecular diagnosis and treatment of colorectal cancer.	Increased risk (overexpression)	(39)
Tumor Specific Antigens	ZEB1	Hypoxic CC tissue (IHC); CC cell lines (*in vitro*)	IHC; qRT-PCR; ChIP; TCGA bioinformatic analysis	Phase I mechanistic case-series + TCGA analysis	Prognostic marker; therapeutic target (hypoxia/TAM axis)	increases CCL8 secretion and TAM recruitment	Increased risk/poor prognosis	(40)
Cancer Cell Proliferation Markers	MSC-F, VEGF, CA 125, and SCC-Ag	Plasma (ELISA) and cervical tissue (IHC)	ELISA; IHC	Phase I case-control	Exploratory ECM/TAM recruitment markers	Diagnosis of both forms of cervical cancer and demonstrated clinical applicability and strong diagnostic efficacy	Increased risk	(41)
	CD44 identification	Cervical tissue	IHC and qPCR	Phase I case-series (n=140; normal, CIN grades, CC)	CIN prognostic subgrouping; triage of CIN for treatment vs. surveillance	Correlates with epithelial-mesenchymal transition (EMT), cellular proliferation, and the estrogen receptor (ER) status. The expression levels of CD44, Ki67, Cas3, and ER serve as biomarkers to differentiate between various groups of cervical intraepithelial neoplasia (CIN) and cancer.	Increased risk (overexpression)	(42)

Phase I, discovery/association; Phase II, validation/clinical performance; Phase III, prospective/RCT-level evidence.

### 2.1 Inflammation-associated biomarkers of CC

#### 2.1.1 Single nucleotide polymorphisms

SNP refers to a genetic variation that occurs at a specific place in the DNA sequence, involving a single nucleotide. SNPs in cytokine genes have been linked to a higher risk of CC (43). Genotyping of host SNPs may yield meaningful biomarkers for CC risk assessment (14). Machine learning-assisted studies of cytokine gene variations and sociodemographic variables can be used to accurately predict the risk of CC (44). CC risk was linked to the *TGFB1* T10C and *XRCC1* G399A SNPs. Patients with the majority allele *TGFB1* T10 (Leu) or the variant allele *XRCC1* 399A (Gln) are 1.5 times more likely to develop CC (14). This polymorphism affects the signal peptide region of TGF-β1, a cytokine with dual roles in CC progression. TGF-β1 is a context-dependent cytokine, and in early stages of tumor, it acts as a tumor suppressor by inhibiting cell proliferation and promoting apoptosis, thereby maintaining cellular homeostasis (45, 46). However, patients carrying this majority have been associated with altered TGF-β1 secretion levels, potentially disrupting this tumor-suppressive function and facilitating HPV persistence and CC progression.

TNF-α is an inflammatory cytokine that is important for the immune response as well as for the progression of cervical lesions. The effect of *TNF-*308α G>A on TNF-α was linked to the risk of CC, indicating the need for additional research in a broader population and multicenter context to demonstrate the efficacy of emerging biomarkers as CC risk stratification biomarkers (15). This polymorphism in *TNF-α* is at the promoter region and is associated altered expression levels (higher). As TNF-α is a pro-inflammatory cytokine it leads to tumor cell survival and progression (47). Several SNPs in cytokines, such as IL-12, IL-6, and IL-10, were found to be possible biomarkers for predicting or diagnosing squamous intraepithelial lesions and CC in multiple studies (48–51). There is a link between the *IL-10* gene polymorphism rs1800896 and increased tissue inflammatory cytokines and an elevated risk of CC (16). This polymorphism is *IL-10* gene is located in the promoter region, affecting IL-10 expression levels (52). In humans, *IL-6* gene SNPs (rs1800795, rs1800796, and rs1800797) are linked to an increased risk of cancer. These polymorphisms alter *IL-6* promoter activity, leading to elevated IL-6 levels that activate the JAK/STAT3 signaling pathway, thereby promoting tumor cell proliferation, apoptosis induction, angiogenesis, and immune evasion (53). These three *IL-6* gene polymorphisms could be tested as population-based, quick, low-cost, PCR-based predictive biomarkers for cancer diagnosis and research (17).

Polymorphisms in miRNA genes can influence miRNA production, which may be linked to cancer formation. rs2297538 and rs2297537 in miR-126; rs1292037 and rs13137 in miR-21; and rs2745709 in miR-221/222 are SNPs that may play a role in the development of CIN or CC (54). In the Han Chinese population, the PTEN 3’UTR rs34140758 locus SNP is linked to the risk of CC. The rs34140758 SNP may influence PTEN gene expression regulation via interactions with the miRNAs hsa-miR-586 and hsa-miR-622 (55). However, the utility of developing biomarkers for risk classification in the management of CC patients requires additional research.

#### 2.1.2 DNA methylation

The widespread use of primary HPV-based screening has prompted researchers to look for a triage test that has high sensitivity for detecting CC and precancer while also increasing specificity to avoid overtreatment (56). FAM19A4 and miR124–2 gene methylation studies have shown potential in the triage of hrHPV-positive women. FAM19A4 is a particular biomarker for malignant cervical tumors. FAM19A4 methylation study could be used as a secondary screening approach for cervical (pre)cancer diagnosis. In HPV-positive women aged 30 years and older, a negative dual methylation test indicates a low risk of CC. This dual methylation of should be considered an objective triage test in HPV-based cervical screening programs (18, 19). Nearly all cervical carcinomas, including unusual histotypes and hrHPV-negative carcinomas, may be detected via methylation studies. According to these findings, a negative methylation assay result of this axis is likely to rule out CC (57). FAM19A4/miR124–2 methylation analysis has good triage performance on baseline screening samples, with cross-sectional CIN3+ sensitivity and long-term triage-negative CIN3+ risk equal to cytology triage appears to be a good and objective alternative to cytology in triage scenarios in HPV-based cervical screening (58). Prospective population-based studies are needed to confirm the diagnostic usefulness of FAM19A4 methylation for cervical (pre)cancer identification (59).

Another study showed that chemokine 13 (CXCL13) and its receptor 5 (CXCR5) play a role in the development of cancer. In CC cell lines and primary tumor tissues, CXCL13 downregulation was linked to hypermethylation. Furthermore, a CpG dinucleotide in the promoter region of CXCL13 near the HIF-1a transcription factor motif was continuously methylated in CC cells and related to HIF-1a (20). CXCL13 hypermethylation is linked with promoter silencing and diminished gene expression in CC. As CXCL13 is expressed on immune cells, its reduced expression prevents immune infiltration to tumor sites suggesting a role in CC progression through loss of chemokine-mediated immune regulation (60).

Binding of the E6-E6AP complex to p53 results in the degradation of p53 via ubiquitination and increased DNA methyltransferase 1 (DNMT1) expression. E7 binds with pRb to release E2F, which also promotes DNMT1 overexpression. The increased activity of DNMT1 hypermethylates the promoters of tumor suppressor genes and silencing of tumor suppressor genes results in cellular transformation and carcinogenesis.

Through the TP53-A3B pathway, 5-Hydroxymethylcytosine (5-hmC), epigenetic DNA modification levels are reduced in CIN2 compared with CIN3. Because A3B may affect genomic stability, the loss of 5hmC may increase the likelihood of mutation and CC progression. As a result, these epigenetic modifications could be utilized for CC risk prediction (61). The transcriptional profile and DNA methylation data reveal the relationship between UBE2V1 and CC progression (62). The potential use of the novel methylation marker hypermethylated PCDHGB7 in cervical scrapings and endometrial brushings for complement hysteroscopy diagnosis by offering a noninvasive method for early CC detection (63).

### 2.2 Immune cell biomarkers

**Figure 3:**
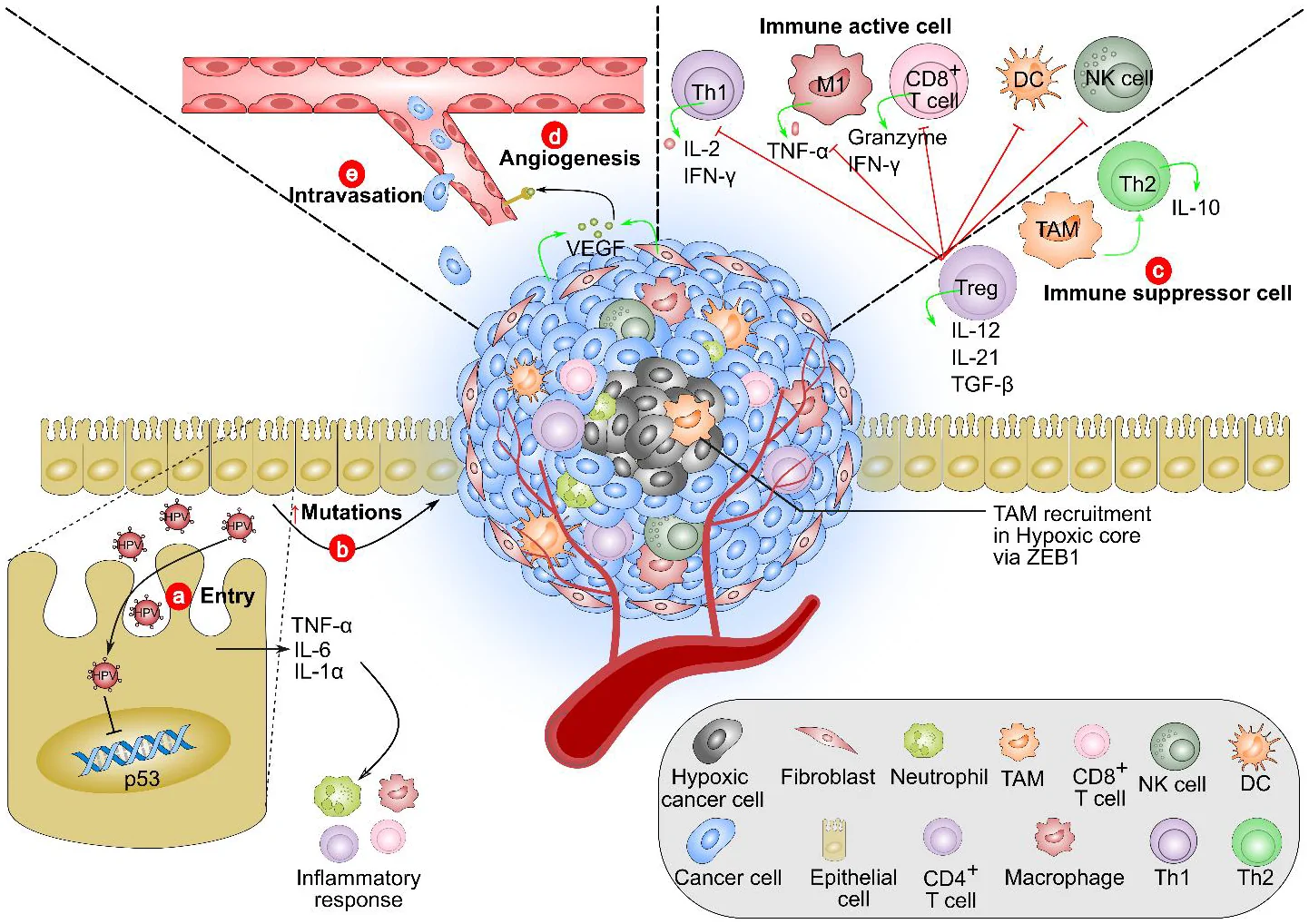
Schematic representation of the tumor microenvironment (TME) in cervical cancer progression. This image illustrates the dynamic processes within the tumor microenvironment (TME) during the progression of cervical cancer. **(a)** Human papillomavirus (HPV) infection: HPV enters epithelial cells of the cervix, where its viral genes interfere with normal cellular functions, including inhibition of p53, leading to the secretion of cytokines and generation of an inflammatory response. **(b)** Accumulation of mutations: Over time, mutations accumulate within the infected epithelial cells, driving the transformation of normal cells into cancerous cells. **(c)** Activation of immune-suppressor cells: immune suppressor cells, including regulatory T cells (Treg), tumor-associated macrophages (TAM), and T-helper 2 cells (Th2), within the TME become activated and secrete cytokines that suppress the activity of immune effector cells. **(d)** Angiogenesis: within the TME, various cell types such as cancer-associated fibroblasts (CAF) and tumor cells secrete angiogenic factors, including vascular endothelial growth factor (VEGF), leading to the generation of new blood vessels. **(e)** Intravasation: as the tumor progresses, cancer cells acquire invasive properties and enter nearby blood vessels. Once in the bloodstream, these cancer cells can disseminate to different body organs, leading to metastasis.

Immune cells play unique and certain antitumor roles; thus, immune cells have potential as biomarkers in different cancer types (64). The extensive crosstalk between tumor cells and immune cells in the tumor microenvironment (TME) (which consists of heterogeneous cell populations, malignant cells, and nonmalignant cells) supports tumor proliferation and metastasis (**Figure 3**). Hence, the TME is an important aspect that manipulates patient prognosis and response to treatment (65). Therefore, investigating the association between tumor progression and immune cells as CC biomarkers for prognosis and treatment response is of utmost importance.

Excessive immune cell infiltration indicates a poor prognosis in CC patients (64, 66). Lymphopenia and an elevated neutrophil-to-lymphocyte ratio were also shown to be strongly associated with poor outcomes, with additional markers suggesting possible connections (67). High numbers of regulatory B cells are observed in CC and have been linked to the promotion of metastasis by reducing perforin- and granzyme B-secreting CD8+ T cells (21).

However, it was also discovered that the presence of tumor-specific CD4 T cells, particularly at the tumor site, is required for immune-mediated tumor rejection (68). Tumor Ag–specific CD4+ T cells are found in greater proportions at tumor sites than in the circulation, suggesting that they are involved in direct or indirect antitumor effector activities *in situ* (69).

The TME plays a critical role in the complex cascade of events leading to the development and progression of cervical cancer, from HPV infection and mutation accumulation to immune suppression, angiogenesis, and ultimately, metastasis to distant organs.

The immunologic interactions involved in the progression and treatment of CC are complicated. Cervical endocervical adenocarcinoma (CESC) develops in an immune microenvironment dominated by invading immune and stromal cells, and a two-gene signature comprising CD1C and CD6 can effectively predict CESC prognosis (70). T cells and mast cells were found to be strongly associated with the survival rate of CC patients among the 22 categories of immune cells studied. Furthermore, a study revealed that the genes BTNL8, CCR7, CD1E, CD6, CD27, CD79A, GRAP2, SP1B, LY9, and CCR5 are linked to survival and that the CXCL9, -10, and -11/CXCR3 axes could be potential targets for CC treatment (71). Mei et al. developed an immune-related four-gene signature for CC prognosis that included RIPOR2, DAAM2, SORBS1, and CXCL8 (72). Similarly, CD45RO+ tumor-infiltrating T cells near the invasive margin and FOXP3+ tumor-infiltrating regulatory T cells in the central tumor area could be valuable biomarkers for CC risk stratification (73). Despite the simultaneous expression of many immune inhibitory genes, patients with tumors expressing genes predictive of T-cell infiltrates (hot tumors) had a better prognosis than did patients with tumors lacking expression of genes associated with T-cell infiltrates (cold tumors), regardless of whether these tumors expressed immune inhibitory genes (74). A deeper understanding of the interplay of immune cells in the tumor microenvironment is crucial for future research regarding their potential role as biomarkers and therapeutic target in CC.

### 2.3 Cytokines

The CC progresses via regional and systemic cytokine functions in host homeostasis (75). An increase in immune cells increases the expression of the proinflammatory cytokines interleukin-12 (IL-12), interferon-γ (IFN-γ), and tumor necrosis factor-α (TNF-α) and decreases the cytokine IL-10 (76). The ratios of CD4+CD25+ regulatory T cells in the peripheral blood and the serum IL-10 concentration are linked to CC activity. Both of these elements are involved in the formation and progression of CC, and they are mutually related and influence one another. Patients with CC who have low IL-10 expression have a better chance of surviving (22).

Chronic genital tract infection with HR-HPV and the Th17 immune response induce chronic inflammation via the generation of IL17 and other proinflammatory cytokines, producing a favorable environment for tumor formation (77). In CC, IL17A and heparanase may increase tumor angiogenesis, cell proliferation, and invasion through the NF-κB signaling pathway (78). The *Nocardia rubra* cell wall skeleton (Nr-CWS) is an immunotherapeutic for HR-HPV infection, and CIN promotes immune function by upregulating T-cell subsets and inhibiting the PD-1/PD-L1 pathway (76). When CC (HPV+) patients were compared to healthy controls, the serum sTREM-1, TNFα, IFNβ, IL-1, and IL-6 levels were considerably greater. The levels of IL-10 and IL-2 followed a similar pattern. These results suggest that systemic inflammation plays a role in the pathogenesis of CC (79). Tumor size, FIGO histological grade, lymph node metastasis, stromal invasion, and tumor differentiation were linked to IL-1 and IL-6 expression. Positive IL-1 and IL-6 expression was found to be substantially linked to a bad prognosis. These factors could be useful prognostic indicators and possible treatment targets for CC (80).

IL-9R is overrepresented in tissue biopsy specimens from CIN patients with CC. In HeLa cell lines, treatment with the Th9 signature cytokines IL-9 and IL-21 decreased proliferation, increased apoptosis, and activated MHC I and E-cadherin expression. As a result of T-cell cytotoxicity, Th9 cells appear to boost the anti-tumor immune response and play a critical role in controlling malignant cell transformation (81). GDF15 (Growth differentiation factor 15) increased CC cell proliferation by upregulating CyclinD1 and CyclinE1 and downregulating p21 via the PI3K/AKT and MAPK/ERK signaling pathways in combination with ErbB2 (82).

In women with reduced IL-1β and IL-18 expression, the probability of preneoplastic lesions progressing to cancer was 2.5 and 2.08 times greater, respectively (23). Both IL-1β and IL-18 are pro-inflammatory cytokines that have a pivotal role in generating an early immune response against tumor cells (83, 84). Reduced expression of both of these cytokines has been linked to increased chances of progression from preneoplastic lesions to CC. Th2 and Th3 cytokines were shown to be more abundant in CC patients than in controls, but IFN-γ, a Th1 cytokine, was found to be more abundant in controls than in CC patients (16). IFN-γ is a key cytokine that mediates antitumor immune response by activating cytotoxic T cells and NK cells. Diminished expression of IFN-γ or dysfunction IFN-γ signaling may diminish immune surveillance (85) and can lead to CC progression and its reduced levels may result in poorer progression. The safety and efficacy of adjuvant immune modulation therapy with the checkpoint inhibitor ipilimumab [anti-CTL antigen-4 (anti-CTLA-4)] after chemoradiation therapy (CRT) for newly diagnosed node-positive human papillomavirus (HPV)-related CC were studied in a phase I clinical trial (GOG-9929). Moreover, TNF-α, IL6, and IL8 levels were found to be substantially linked with poorer progression-free survival after CRT (86). High-grade intraepithelial lesions and invasive CC have been linked to persistent infection with high-risk HPV strains. Two key proinflammatory cytokines, IL-2 and IL-23, are downregulated in cervical biopsy specimens from HPV lesions taken at the lesion site (87).

### 2.3 Non-coding RNAs

#### 2.3.1 MicroRNAs

miRNAs are functionally engaged in multiple major hallmarks of cancer in different stages of CC, including evasion of growth suppressors, evasion of replicative immortality, activation of invasion and metastasis, resistance to cell death, and prolonged proliferative signaling (88). The E7 oncoprotein of hrHPV appears to be a key component for cell immortalization and transformation, affecting crucial processes such as cell proliferation, apoptosis, and the immune response. hrHPVs play a critical role in changing the microRNA (miRNA) profile in chronic viral infections (89). These dysregulated microRNAs may play a key role in the progression of CC. Some stage-specific microRNAs can be employed as biomarkers for cancer classification and tracking CC progression due to the substantial dysregulation of miRNAs in cervical carcinogenesis, as they are associated with dynamic changes in expression and the ability to detect viral persistence, risk prediction of low-grade lesions CIN 2 to CIN 3, and cancer progression (25). The ability of these methods to distinguish High-Grade Squamous Intraepithelial Lesion (HSILs) from nondysplastic lesions should help with early detection and reduce overtreatment of otherwise regressive early preinvasive lesions (90).

Circulating microRNAs may be useful molecular markers for early follow-up, according to previous research (91). The severity of cervical lesions can be assessed noninvasively by monitoring the plasma levels of miR-21, miR-214, miR-34a, and miR-200a. Moreover, the plasma profile of these miRNAs can be used to significantly differentiate CIN1- patients from CIN2+ patients (92). The presence of miR-34a in HPV infection, particularly in HPV16 infection, may be linked to the onset and progression of CC (24). In liquid-based cytology samples, four miRNAs (miR-205-5p, miR-130a-3p, miR-4531, and miR-381-3p) might be employed as biomarkers for CIN 3 (25, 26). miRNAs such as miR-638, miR-521, miR-210, miR-20 and miR-515 have been shown to be useful biomarkers for distinguishing normal individuals, CIN patients and cancer patients (93–95). Distinct subtypes of infection may activate different processes in disease; miRNAs play complex roles in carcinogenesis, and there may be several targets for which multiple mechanisms are involved. Upregulation of lysine-specific demethylase 5A by the human papillomavirus type 16 E7 oncoprotein enhances CC progression by modulating the microRNA-424-5p/suppressor of the zeste 12 pathway (96).

MicroRNA-155 was found to be overexpressed in the peripheral blood and CC tissues of CC patients compared to healthy controls. MicroRNA-155, through reducing SOCS1 expression and promoting a Th17/Treg imbalance, plays a role in the onset and progression of CC (27, 28). Additionally, it facilitates autophagy via the mTOR pathway and decreases PDK1 expression (97). Additionally, miR-92a-5p and miR-155-5p are increased in HPV-infected low-grade squamous intraepithelial lesions (LSILs) and high-grade squamous intraepithelial lesions (HSILs); these two miRNAs can be used as prognostic biomarkers for CC patient prognosis (30).

Compared to that in nearby normal tissues, miR-34b expression was reportedly downregulated in CC in one study. MiR-34b and TGF-1 are possible targets for CC therapies because they contribute to cell proliferation and death (98). The expression of miR-34a-5p and miR-218-5p was found to be an independent predictor of overall survival in CC patients. A recent study suggested that assessing the expression of these particular miRNAs in noninvasive urine samples could lead to reliable identification of biomarkers for early CC diagnosis and prognosis (29). The changes in miR-34a and miR-15b expression levels with the progression of CC confirm that they are molecular biomarkers of CC (89). In the serum of SCC patients, miR-15b was overexpressed, while miR-34a and miR-218 were under expressed. The mRNA expression levels of several selected gene targets [Cyclin D1 (CCND1), Cyclin E1 (CCNE1), B-cell lymphoma 2 (Bcl-2) and MutS homolog 2 (MSH-2)] were also assessed.

The six circulating miRNAs (hsa-miR-26b-5p, hsa-miR-146b-5p, hsa-miR-191-5p, hsa-miR-484, hsa-miR-574-3p, and hsa-miR-625-3p) were linked to cervical lesions and could be used as noninvasive biomarkers in the future (31). In CC patients, the serum miR-100 concentration could be a valuable diagnostic biomarker for predicting lymph node metastasis and disease-free survival (99). A miRNA-based signature including serum miR-21, miR-125b, and miR-370 appears promising for early-stage CC diagnosis and prognostic prediction (100). Increased expression of the miRNA signatures miR-146a-5p, miR-151a-3p, miR-2110, and miR-21-5p in plasma samples of CC patients was reported to be a promising novel biomarker for the diagnosis of CC (101).

miRNA and protein feedback loops promote the proliferative and metastatic capacities of CC cells. Several findings have suggested multiple miRNA and protein loops in CC that could be promising biomarkers for CC diagnosis and treatment. For instance, VEGF-A and miRNAs such as miR-15a-5p, miR-16-5p, miR-29a-3p, miR-195-5p, and miR-205-5p, which have multiple binding sites on VEGF-A, are highly expressed in the serum of CC patients with venous thromboembolism; they can also act as potential biomarkers and have potential for use in the treatment of venous thromboembolism and CC (102). Inhibition of miR-25 decreased HeLa cell growth and motility while increasing KLF4 protein levels. Inhibition of miR-25 may increase the expression of KLF4, which results in increased cell proliferation and growth factor (CC) motility (103). BCDIN3D reverts the expression of miR-195-3p, which may impede CC cell proliferation. After several animal assays and clinical tests, miR-195-3p mimics/BCDIN3D siRNA may be used to treat CC in the future (104). RNA-seq data from squamous cell carcinoma samples suggest that platinum-based first-line treatment can be influenced by miRNAs. Additionally, certain miRNAs were shown to be associated with the response to platinum-based therapy in numerous malignancies (105). Decreased expression of miR-200a, miR-200b, and miR-429 predicts recurrence and tumor radio resistance, regardless of the clinical marker and hypoxia (106). In CC development, the BBOX1-AS1-hsa-miR-125b-5p/hsa-miR-125a-5p-CDKN2A ceRNA network is quite useful. The downregulated miRNAs hsa-miR-125b-5p and hsa-miR-125a-5p, which are targeted by the upregulated lncRNA BBOX1-AS1, target the upregulated cyclin-dependent kinase inhibitor 2A gene (CDKN2A) (107). Exosomal miR-125a-5p could be used as a biomarker for diagnosing CC (108). In addition, numerous studies suggest that different miRNAs, such as miR-27a, miR-1228-3p, miR-1468-5p, miR-885-5p, miR-455-5p, miR-143-3p, miR-192-5p, miR-193b-3p, miR-378a-3p and miR-215-5p, could be used as prognostic and therapeutic targets in the treatment of CC (109–116).

#### 2.3.2 lncRNA

CC is one of the most likely causes of cancer death in women. Moreover, mRNA–miRNA-lncRNA signatures help physicians improve the prognosis of individuals (117). The miR-192-5p/HNF1A-AS1/VIL1 panel, discovered by Xu et al, correctly distinguishes cancer from normal cervix (118). Due to its poor prognosis and high mortality rate, dysregulated lncRNAs/miRNAs, such as LINC00925/miR-9 and MIR155HG/miR-155, cause CC by building an interaction network with mRNAs, emphasizing the necessity of understanding the underlying pathways of ncRNAs in CC development (119). The oncoprotein YAP1 is inhibited by Small Nucleolar RNA Host Gene 3 (SNHG3), which also influences the transcription of multiple YAP1 target genes. Thus, SNHG3 could be used as a prognostic biomarker and a therapeutic target for this disease (32). ABHD11 antisense RNA 1 (ABHD11-AS1) aided in CC carcinogenesis by competitively trapping miR-330-5p to upregulate MARK2, suggesting that ABHD11-AS1 could be used as a CC biomarker (33). The lncRNA ATB is linked to malignant phenotypes and a poor prognosis in CC patients. ATB could be a useful prognostic and therapeutic target for CC patients (120). The lncRNA SPINT1-AS1 has been identified to inhibit miR-214 production while stimulating Wnt signaling, indicating its potential as a prognostic biomarker and therapeutic target for CC (34). HOTAIR binds to miR-214-3p and may function as an enhancer RNA, promoting cell proliferation and inhibiting apoptosis in HPV16-positive CC cells. The HOTAIR/miR-214-3p/Wnt signaling pathway may play a key role in HPV16-positive CC regulation (121). However, the role of miR-21 in cervical lesions is unclear. miR-214 plays a role in CC progression. In patients with HPV infection, miR-let-7a may be increased at a systemic level (122). XIST plays a key role in HPV-mediated carcinogenesis, suggesting that HPV16- and HPV18-associated cancers have different molecular signatures. Furthermore, various XIST-related axes, such as XIST | miR-23a-3p | LGR4 and XIST | miR-30b-5p or miR-30c-5p or miR-30e-5p I ADAM9, were found to have a substantial impact on the overall survival of HPV16 and HPV18CESC patients, respectively (123).To regulate VDAC1 expression, MEOX2 antisense RNA 1 (MEOX2-AS1) sponged miR-143-3p. Furthermore, a miR-143-3p inhibitor abrogated the antiproliferative and antimetastatic effects of MEOX2-AS1 knockdown. The MEOX2-AS1/miR-143-3p/VDAC1 pathway is involved in the progression of CC, suggesting that this pathway is a unique therapeutic target for CC treatment (124). Vaginal microbial dysbiosis and increased serum levels of miR-18a and PD-L1 are found in HPV-positive CC patients. For detecting HPV-positive CC, miR-18a and PD-L1 have diagnostic significance (125). HPV infection is linked to aberrant lncRNA deleted in lymphocytic leukemia 1 (DLEU1) expression, which could be used as a diagnostic and prognostic biomarker for HPV+ individuals with CC. 2022) (126). DARS-AS1 knockdown inhibited the development of CC cells by sponging miR-188-5p and downregulating HMGB1. As a result, DARS-AS1 could be a possible target for CC treatment (127). By targeting miR-27a-3p to upregulate BTG2 expression, LINC01089 acts as a tumor suppressor, preventing the development of CC (128). HOXD cluster antisense RNA 1 (HOXD-AS1), an oncogenic lncRNA, operates as a tumor-promoting lncRNA in CC via the miR-877-3p/FGF2 axis. HOXD-AS1 could be an efficient treatment target for CC, and a new predictive biomarker (129). The RE-1 silencing transcription factor (REST) has been shown to reduce NPPA-AS1 transcription in CC. In CC, NPPA-AS1 acts as a competitive endogenous RNA by sponging miR-302e and upregulating the expression of Dickkopf-1 (DKK1, a Wnt pathway inhibitor) (130). REST-repressed NPPA-AS1 modulates the miR-302e/DKK1/Wnt/β-catenin signaling pathway to regulate CC development (130). The endogenous sponge FOXD3 antisense RNA 1 (FOXD3-AS1) operates as an endogenous sponge, directly binding and suppressing miR-296-5p (131). In CC, RP11-284F21.9 acts as a tumor suppressor and regulates PPWD1 expression by competitively binding to miR-769-3p, suggesting that the RP11-284F21.9/miR-769-3p/PPWD1 axis could be a viable prognostic biomarker and therapeutic target (132). By sponging miR-519e-5p and eliminating its tumor-suppressive function, GABPB1-AS1 acts as a competitive endogenous RNA (ceRNA), consequently decreasing the expression of Notch2, a well-known oncogene. GABPB1-AS1 has been linked to CC progression and could be a suitable biomarker or target for CC treatment (133).

LINC00116 plays a role in CC carcinogenesis through modulating the miR-106a/c-Jun axis. (2020, Lai et al.) Through the miR-625-5p/LRRC8E axis, LINC00958 promotes CC progression and metastasis (134). Through the miR-625-5p/LRRC8E axis, LINC00958 promotes CC progression and metastasis (135). In CC, knocking down the lncRNA CASC15 hindered cell invasion and the epithelial–mesenchymal transition (EMT) signaling pathway by upregulating E-cadherin expression while downregulating N-cadherin expression. (136). NR2F1-AS1 inhibits CSCC cell invasion and migration through regulating the miR-17/SIK1 axis (137). The activation of miR-124-3p/STAT3/MICA-mediated NKT cell tolerance by LINC00240 increased CC development. (138).

#### 2.3.3 Circular RNAs

Circular RNAs (circRNAs) play a significant role in a variety of physiological and pathological processes, and researchers have discovered new CSCC diagnostic and therapeutic biomarkers as well as the underlying mechanisms (139–141). CXCL8 expression is increased in low-risk tumor tissue and high-risk tumor tissue compared with normal tissue. CirRNAs such as hsa circ 0025721 may upregulate CXCL8 by sponging miRNAs such as hsa-miR-4428, according to RNA-seq and bioinformatics (35).

hsa circ 0107593 binds to and sponges hsa-miR-20a-5p, hsa-miR-93-5p, and hsa-miR-106b-5p. hsa circ 0107593 inhibits tumor growth in CC by physically binding to hsa-miR-20a-5p, hsa-miR-93-5p, and hsa-miR-106b-5p (142). Loss-of-function tests *in vivo* and *in vitro* were performed by Wu et al. to investigate the impact of circEPSTI1 expression on CC proliferation. CircumEPSTI1 upregulates SLC7A11 expression by sponging miR-375, miR-409-3p, and miR-515-5p (143). Hu et al. constructed a regulatory network that included two circRNAs, two miRNAs, 22 mRNAs, and 19 drugs/chemicals, and the results revealed new information on the prognosis and treatment of CSCC (144). The circRNA PVT1 can cause CC cells to undergo EMT by targeting miR-1286 through the exosome pathway, which could be a distinct mode of CC progression (145). circNRIP1 regulates the PTP4A1/ERK1/2 pathway, at least in part, by sponging miR-629-3p and further influencing the PTP4A1/ERK1/2 pathway. As a result, circNRIP1 could be used as a predictive biomarker and therapeutic target in patients with CC (36). The human papillomavirus status of patients, which is the most important factor in the development of CC, was found to be significantly correlated with the miRNA markers hsa-miR-26b-5p, hsa-miR-191-5p, and hsa-miR-143-3p, allowing cervical mucus-based testing for cervical squamous cell carcinoma preneoplastic stages (146).

*In vitro* and *in vivo*, miR-135a causes the creation of a subpopulation of cells with CSC features, and the Wnt/β-catenin signaling pathway is required to sustain its tumorigenicity (147). Circ 0000388 was discovered to be a new carcinogenic circRNA in CC that accelerates cancer progression by controlling miR-337-3p and TCF12 and may be employed as a diagnostic biomarker and therapeutic target in the future (37). circAGFG1 sponges miR-370-3p to regulate RAF1 and enhances cancer growth and metastasis by upregulating the RAF/MEK/ERK pathway (148). circZFR binds to SSBP1 and promotes the formation of CDK2/cyclin E1 complexes. CDK2/cyclin E1 complex activation causes p-Rb phosphorylation, leading to the release of activated E2F1 and cell proliferation. circZFR is a novel onco-circRNA that could be used as a biomarker and therapeutic target for CC patients (38).

### 2.4 Hormones and enzymes

CCs can be detected by various molecular techniques, such as PCR, the visual acetic acid method, the DNA Hybrid II test, liquid-based cytology, Pap smear techniques, and colposcopy techniques. Changes in the expression of certain enzymes and hormones, such as insulin and triglycerides, and increased activity of adiponectin may cause CC. Researchers are studying a variety of hormones and proteins to understand and evaluate CC carcinogenesis and management strategies (149). For instance, CC growth and metastasis can be controlled by SOX proteins. SOX transcription factors govern the response of CC cells to chemotherapy and radiotherapy. WNT signaling, the EMT, and the Hedgehog pathway were identified as SOX protein downstream targets. Upstream mediators such as microRNAs, lncRNAs, and circRNAs can also control SOX expression in CC (150). Additionally, APOA1, APOE, and CLU protein expression in the plasma of patients with various stages of cervical lesions was verified by ELISA (151). TYMS (Thymidylate Synthetase) have the potential to be used as CC biomarker since it plays a critical role in the prognosis and course of the disease. The miR-197-3p/TYMS axis can control the degradation of CC cells, laying the groundwork for CC molecular diagnosis and treatment (39).

A novel method using tumor-activatable minicircle (MC) plasmids, which trace and diagnose cancerous lesions by encoding a combination of blood-based biomarkers (Gaussia Luciferase [GLuc], a secreted biomarker that can be easily assayed in blood samples] and imaging reporter genes [Herpes Simplex Virus Type 1 Thymidine Kinase mutant [HSV-1 sr39TK]) to tumor cells, was developed. This study explored ways to overcome the limitations of early cancer detection systems that rely on endogenous blood-based biomarkers and molecular imaging, as well as the transport capabilities and limitations of EVs (152).

*In vitro* and *in vivo*, miR-9-5p has been shown to increase the proliferation and invasion of CC cells. By targeting SOCS5, miR-9-5p may influence angiogenesis and radiosensitivity in CC cells (153). A novel prognostic factor and treatment target for 7-HPV-related cervical SCC is miR143-3p, which targets the mRNA expression of the baculoviral inhibitor of apoptosis protein (IAP) repeat-containing 2 (BIRC2) protein (111). FABP5-induced LNM occurs via a metabolism-dependent mechanism. Furthermore, under hypoxia, miR-144-3p can control FABP5 expression and biological function (154).

The E3 ubiquitin ligase RAD18 has been identified as an oncoprotein with prometastatic features in a variety of cancers; however, its role in cervical carcinoma (CC) is unknown. Silencing RAD18 changed the expression profile of proinflammatory mediators, including interleukin 1 (IL1). Exogenous IL1 therapy also prevented RAD18-mediated CC cell invasion. These findings revealed an underlying mechanism by which RAD18 promotes CC progression, suggesting that RAD18 could be used as a biomarker and therapeutic target in the treatment of malignant CC (155). OTUD5 increases the expression of WD repeat domain 45 (WDR45), ubiquitin-specific peptidase 11 (USP11), GRIP1-associated protein 1 (GRIPAP1), and RNA binding motif protein 10, which are members of the ovarian tumor protease (OTU) family (RBM10). hsa-mir-137, hsa-mir-1913, hsa-mir-937, hsa-mir-607, hsa-mir-3149, and hsa-mir-144 may also suppress OTUD5 expression. They are also involved in Hippo signaling, insulin signaling, the EMT, histone methylation, and phosphorylation kinase binding (156). Through the overexpression of Wnt5a, histone deacetylase 6 (HDAC6) reduces miR-199a transcription and accelerates the progression of HPV-positive CC (157). Ubiquitin-specific protease 18 (USP18), also known as UBP43, is a deubiquitinating (DUB) enzyme that has been associated with numerous human cancers. In CC, USP18 is an oncogenic gene that could be exploited as a biomarker (158).

The MCM3 (minichromosome maintenance complex 3) is required for DNA replication and cell cycle advancement (159). However, bioinformatics analysis is currently limited, and clinical trials are needed to further investigate the role of MCM3 in the progression of CC (160). Five mRNAs, namely, DNMT1, CHAF1B, CHAF1A, MCM2, and kinetochore-related protein 1 (KNTC1), have been identified as potential CC biomarkers (161). The practicality of assessing p16INK4a in fresh cervical tissue samples using an ELISA, as well as its potential as a supplement to existing screening measures for detecting high-grade dysplasia while avoiding colonoscopic referrals, is also important (162). A panel of biomarkers, including TOP2A/MCM2, p16INK4a, and cyclin E1, can be used to identify cervical lesions associated with a greater risk of CC; this can be performed inexpensively in a single noninvasive liquid-based cytology sample (163).

Increased serum Tie-1 levels in CC patients are linked to tumor development and a poor prognosis (164). Cancer cells release nuclear transport proteins, which are detected in the serum. Nuclear transport proteins can potentially be used as potential markers for CCs (165). Multiple serum indicators may increase the accuracy of noninvasive CC detection and may serve as a new liquid biopsy technique for early-stage CC detection (166–170).

After laparoscopic general anesthesia for CC treatment, the levels of C-reactive protein (CRP), procalcitonin (PCT), and triggering receptor expressed on myeloid cells 1 (TREM-1) are abnormally elevated in individuals with pulmonary infection. Their combined detection can be used to forecast the incidence of lung infections in the early stages, and their level should guide appropriate management to improve patient prognosis (171). MMP15 expression has been established in human CC, and it is associated with decreased overall survival and is linked to the expression of prokineticin 2 (PROK2). PROK2 is a novel and useful biomarker for clinical application due to its PROK2’s oncogenic capabilities as a therapeutic target for CC (172). A set of 22 antibodies accurately predicts survival in two different groups of CC patients (173). Integrating ER/PR testing into clinical risk stratification may help patients with Grade I-II EEA have a better prognosis (174, 175). By finding high hCG staining solely in SCC instances, researchers were able to link ectopic hCG expression to cancer development (176). The upregulation of cancer-related genes not previously reported in the context of CC included genes encoding glycerophosphodiester phosphodiesterase domain containing 3, interleukin 1 receptor type II, natriuretic peptide type C, MGAT4 family member C, lecithin-retinol acyltransferase (phosphatidylcholine-retinol-O-acyltransferase), and glucoside xylosyltransferase. Upregulation of the serine (or cysteine) peptidase inhibitor clade B member 9 gene was found to be inversely proportional to granzyme gene family expression. The inhibition of the granzyme B pathway could be a novel strategy for cancer cell immune evasion (177).

EFNA1 could be used to predict the prognosis of CC patients. EFNA1 was found to be involved in a variety of cancer processes, protein creation, and viral generation according to gene ontology and pathway analyses. These findings suggested that EFNA1 could be a predictive biomarker for CC as well as a possible therapeutic target (178). TOP2A overexpression is linked to the grade of cervical intraepithelial neoplasia; however, it does not predict CC prognosis. Similarly, p16 expression is linked to histological dysplasia and malignancy, implying that it has prognostic and predictive utility in the treatment of cervical malignancies (179).

When exposed to cisplatin, FGF13 modulates the expression and distribution of hCTR1 and ATP7A in cancer cells, reduces platinum influx, and enhances platinum sequestration and efflux. We discovered that FGF13-mediated platinum resistance is dependent on the microtubule-stabilizing action of FGF13. The platinum resistance effect was only induced by overexpression of FGF13 with the -SMIYRQQQ- tubulin-binding domain. the mechanism of FGF13-induced platinum drug resistance, and it shows that FGF13 could be used as a chemotherapeutic sensitization target and prognostic biomarker (180). PLOD2 has been linked to a poor outcome in CESC patients and could be used as a new diagnostic and prognostic marker (181). CCT6A (chaperonin-containing tailless complex polypeptide subunit 6A) is a newly discovered clinical biomarker for many malignancies. CCT6A expression is higher in tumor tissues and is linked to increased tumor size, increased lymph node metastasis, increased FIGO stage, and a worse prognosis in CC patients (182). Rspo1 and Slit2 levels are strongly related to patient tolerance. Rspo1 and Slit2 levels, on the other hand, were negatively related to the morbidity score of patients receiving CT and/or RT. As a result, Rspo1 and Slit2 may be possible predictive biomarkers for patients with CC who receive CT or RT after surgery, which supports the ongoing investigation of the therapeutic value of Rspo1 and Slit2 (183). CD155 forms a complex with AKT, activating the AKT/mTOR/NF-B pathway and inhibiting autophagy and apoptosis. CD155 could be used as a CC screening and therapeutic biomarker (184). The immunohistochemistry (IHC) results for PD-L1, PARP1, and MMRs showed that patients with NECC should be treated with immunotargeted agents. Immune checkpoint therapy was successful in patients with PD-L1 positivity and MMR loss in patient. More data should be collected, and effective biomarkers must still be discovered based on clinical feasibility (185). PARP1 is a pancancer predictive biomarker and is linked to the status of immunotherapy-associated signatures, suggesting that it could be a viable biomarker for predicting ICI response in a variety of malignancies (186).

Williams syndrome transcription factor (WSTF) is abundantly expressed in CC tissues and cells, and by stimulating the PI3K/Akt signaling pathway, downregulation of WSTF can decrease CC cell proliferation, invasion, and migration. WSTF is a promising novel biomarker for colorectal cancer (187). A 15-gene classifier may be a useful tool for guiding treatment decisions for locally advanced CC (188). CDC7 plays a critical role in the progression of CC. The use of this biomarker could aid in the early detection and treatment of CC (189).

By modulating lipid metabolism, HSDL2 plays a role in oncogenesis and leads to poor prognosis in CC patients. Based on new information on HSDL2 functions, which could lead to the development of a biomarker for prognosis and a new therapeutic target (190). Many malignancies have been observed to have uncontrolled neutrophil gelatinase-associated lipocalin (NGAL), which suppresses tumor suppressor genes, including p53, leading to cell proliferation. Because therapy time and modality are both significant in CC and a shorter treatment duration is related to better results and lower NGAL levels, NGAL could prove to be a valuable biomarker, albeit further research is needed to back up this assertion (191). Increased ADAM12 expression in CC can be used as a prognostic indicator (192). The somatic mutation status is a predictive indicator of PLN metastasis in early-stage CC and, as a result, is associated with a poor prognosis. As a result, rather than a single genetic mutation, comprehensive genetic mutations should be studied extensively to uncover novel genetic markers with therapeutic utility (193). In circulation, sPlGF and sFlt-1 could be useful diagnostic biomarkers for CC, and their usage in combination could be even more useful in diagnosing individuals with early CC (194). AKR1C1 and TKTL1 showed promise as biomarkers for LSIL diagnosis in Mexican women, and their sensitivity and specificity are comparable to those of biomarkers used in clinical trials (195). The KRASmt/SMAD4mt signature may be a predictor of radio resistance, and carbon ion radiation may be a treatment option for early-stage cervical malignancies (196).

Through a mechanism that includes thiol-mediated oxidative stress, PI3K-activated CC cells are sensitive to glutamine deprivation. These findings support the use of telaglenastat for glutamine-dependent radioresistant cervical malignancies and show that alterations in the PI3K pathway can be exploited as a telaglenastat sensitivity indicator (197). Methyltransferase-like 3 (METTL3) is a cancer oncogene that belongs to the m6A methyltransferase family. The enzymes that control m6A epitranscriptomic alterations regulate innate immunity in the host. High levels of METTL3 are associated with CD33+ MDSC growth, and METTL3 and CD33+ MDSCs are independent prognostic variables in CC (198). TOP2A and CENPF are MRs that are activated and overexpressed in CC. High expression of these genes is linked to various prognostic and molecular aspects (e.g., somatic mutations) and is significantly greater in CC than in many other cancer types. These compounds could be useful indicators and anticancer therapeutic targets in the case of CC (199). Nurr1 plays unexpected roles in promoting tumor growth and radio resistance and can be used as a therapeutic target for CC treatment (200). RRM2 is anticipated to become a new potential CC diagnostic and prognostic biomarker, providing evidence to support the study of CC mechanisms (201). KDM5A binds with the miR-424-5p promoter, suppresses its tumor-suppressive function and acts as a tumor activator (96). Hepika is an enzyme-linked immunosorbent test (ELISA) that targets the E6, and p53 and E7 and pRb protein complexes. The Hepika test was discovered to be a reliable biomarker for HPV-related CC. Yet, Further clinical trials are warranted to confirm its efficacy as a screening test for differentially detecting CIN and CC patients (202).

### 2.5 Tumor specific antigens

Hypoxia-induced ZEB1 plays an unexpected role in cancer progression by nurturing a prometastatic environment through steadily increasing CCL8 secretion and TAM recruitment. As a result, ZEB1 could be used as a candidate biomarker for tumor progression and targeted to disrupt hypoxia-mediated TME refurbishment (40). M-CSF, matrix metalloproteinase-2 (MMP-2) and the specific tissue inhibitor TIMP-2 may play a role in cancer development (203, 204). In patients with CC, high-grade CIN3, ectropion, plasma levels and tissue expression of macrophage colony-stimulating factor (M-CSF), vascular endothelial growth factor (VEGF), matrix metalloproteinases (MMP)-2 and MMP9, and their tissue inhibitors TIMP1 and TIMP2 were measured. Compared to those in the ectropionic and CIN3 groups, the epithelial expression of M-CSF and TIMP1 in cancer tissue was markedly greater. All of these proteins may play a role in CC diagnosis (41). M-CSF (macrophage colony-stimulating factor), matrix metalloproteinase-2 (MMP-2) and a specific tissue inhibitor (TIMP-2) may all play a role in cancer disease development. Based on ROC analysis, M-CSF appears to be the best option for cancer diagnosis at all stages of the disease (203). Overexpression of the Rh family C glycoprotein (RHCG) inhibited CC cell proliferation and migration, as demonstrated by decreased expression of transforming growth factor (TGF)-1, matrix metalloproteinase (MMP)-2, and MMP-9 in cancer cells (205). In addition to these molecular markers, classical serum protein biomarkers such as squamous cell carcinoma antigen (SCC-Ag) and carcinoembryonic antigen (CEA) remain clinically relevant, with elevated levels observed in CC patients compared to controls and lower levels in CIN (206).

CUE-101 is a new therapeutic fusion protein that selectively activates and expands HPV16 E711-20-specific CD8+ T cells as an off-the-shelf therapy for the management of HPV16-driven tumors such as HNSCC, CC, and anal tumors. In an ongoing phase I trial, CUE-101 showed selective growth of an HPV16 E711-20-specific population of cytotoxic CD8+ T cells, a favorable safety profile, and *in vitro* and *in vivo* evidence supporting its clinical efficacy (207) In CIN-1 patients, persistent infection with HR-HPV, primarily HPV-16, may enhance the expression of CD39 and CD73 in precursor lesions through the synthesis of TGF-β, resulting in an immunosuppressive milieu and allowing progression to CC. CD39 and CD73, two ectonucleotidases involved in the synthesis of adenosine (Ado), which suppresses particular antitumor immune responses, are expressed in CC precursor lesions (208). C glycoprotein (RHCG) is a member of the rhesus (Rh) family that was first discovered as a blood-type antigen in Rh. Overexpression of RHCG inhibited cervical tumor growth. As a result, RHCG could be used as a predictive biomarker and therapeutic target in the treatment of human CC (205).

The canonical pathways that are overrepresented in cervical squamous cell carcinoma (CSCC) include acute-phase response signaling, JAK/Stat signaling, and IL-4 signaling, among others (151). The presence of tumor-associated macrophages in the hypoxic tumor microenvironment (TME) has been linked to cancer progression. Hypoxia-induced ZEB1 plays an unexpected role in cancer progression by fostering a prometastatic environment through increased CCL8 secretion and TAM recruitment. As a result, ZEB1 could be used as a candidate biomarker for tumor progression and a target for disrupting hypoxia-mediated TME remodeling (40).

HPV E7 cfDNA represents a possible tumor marker with great specificity and moderate sensitivity. Furthermore, in resource-limited situations, the RPA-LF test provides an inexpensive, fast, and ultrasensitive technique for identifying HPV cfDNA (209).

### 2.6 Cancer cell proliferation markers

Squamous cell carcinoma of the cervical mucosa (CSCC) is one of the most frequent gynecological cancers. The prognosis for CSCC varies because it is a physiologically heterogeneous disease. As a result, developing predictive biomarkers that represent the biological heterogeneity of the disease could lead to more effective interventions for individuals with a poor prognosis. In CSCC, 149 immune genes were found to be predictive of overall survival. These findings provide researchers with a better understanding of immune genes in the tumor microenvironment, as well as a list of prognostic immunity genes in CSCC. The 384 intersecting genes were mostly enriched for T-cell activation, membrane region, carbohydrate binding, and cytokine–cytokine receptor interaction according to differential expression analysis, Gene Ontology, and Kyoto Encyclopedia of Genes and Genomes studies (206). Macrophage colony stimulating factor (M-CSF) and VEGF activation are thought to play a role in cancer pathogenesis and metastasis. The combination of MSC-F, VEGF, CA 125, and SCC-Ag for the diagnosis of both forms of CC was found to have clinical application and good diagnostic power in a previous study (41).

Interrupting locoregional immune suppression in CC and controlling metastatic dissemination to the TDLN via local PD-(L)1 inhibition. Furthermore, findings suggest that CD8+FoxP3+CD25+ T cells as potential therapeutic targets and biomarkers for PD-(L)1 checkpoint blockade (210). In combination with hr-HPV testing, genomic DNA methylation, particularly of the tumor suppressor gene CADM1, may play a role in detecting histologic HSIL+ lesions (211). The lack of CD34+ fibrocytes inside the stroma has been linked to invasive carcinomas in several investigations. The stroma of the normal cervical stroma and the stroma close to cervical intraepithelial lesions are densely packed with CD34+ fibrocytes, while the stroma of invasive carcinoma is almost devoid of this cell type. Squamous carcinoma stromal invasion was characterized by a localized loss of CD34+ fibrocytes. This approach can be utilized to detect small foci of stromal invasion in the early stages of malignancy (212). With the advancement of cervical intraepithelial neoplasia, the use of a stem cell index based on CD44 identification has increased dramatically. Furthermore, at both the protein and mRNA levels, CD44 expression is associated with EMT, proliferation and the ER state. Two, the expression of CD44, Ki67, Cas3, and ER can be used to distinguish between groups of CINs and cancer (42). In CSCC, cancer-associated myofibroblasts (myCAFs) have been identified are associated with poor prognosis. myCAfs shape the tumor extracellular environment, which may help create better cancer treatments. ACTA2, FAP, ITGB4, and POSTN serve as suitable marker genes for distinguishing myCAFs in CSCC, with POSTN exhibiting the greatest specificity and prognostic significance. High expression of POSTN alone was strongly related with lower survival, suggesting it could be a reliable molecular and functional indication of myCAFs and thus of CSCC (213).

### 2.7 Biomarkers for immunotherapy response in cervical cancer

Immunotherapy is an established therapeutic approach for CC and has significantly improved the prognosis of patients with advanced and recurrent CC. PD-L1 is an established immune- related biomarker utilized for Immune Checkpoint Inhibitors (ICIs) therapy in CC. PD-L1 positivity is common in CC, reported in up to 90% of tumors, and is linked with a favorable prognosis, which helps explain why it became the leading biomarker for PD-1/PD-L1 therapy in this disease (214). In clinical trial NCT03816553 (n=37), various immune-related biomarkers were identified in response to anti-PD-1 combination therapy, which included increased CD8+ T cell and their infiltration, PD-L1+ checkpoint ligand proximity (within 20–45 micrometers) at the tumor invasive margin, and increased PANCK^−^PD-L1^+^ immune cells as predictors/biomarkers of anti-PD-1 response and progression-free survival, using multi-parameter spatial immune profiling via multispectral imaging (215). CTLA4, an important immune checkpoint, may also serve as a biomarker for immunotherapy, as it is found positive in 80% of patients with CC (216). RNA expression of CTLA-4 was higher and correlated with high PD1, PD-L2, and LAG3 expression. High CTLA-4 and PD-L1 expression were associated with a favorable prognosis in patients receiving ICI therapy (216). In one study, peripheral blood biomarkers, including pre-treatment neutrophil-to-lymphocyte ratio (NLR), were analyzed in 282 advanced CC patients receiving anti-PD-1/PD-L1 therapy, with low-NLR + PD-L1+ status identifying patients most likely to benefit from checkpoint inhibition (217). This NLR ratio may offer practical cost-effective patient stratification for anti-PD-1/PD-L1 therapy, as a lower NLR (<3.91) predicts superior progression-free survival and overall survival (217). However, more research is required to determine pre-treatment NLR as a biomarker for anti-PD-1/PD-L1 therapy.

In one study of 50 patients with stage III-IVa or recurrent CC undergoing immunotherapy in conjunction with chemo/radiotherapy, blood-based biomarkers were identified, including elevated CD4+ and CD8+ cell counts post-treatment, decreased PD-1 expression, and declining tumor antigens (CA125/SCCA), and were correlated with an increased probability of immunotherapy efficacy (218). Targeted proteomics identified multiple biomarkers consisting of a 5-protein signature, namely ITGB5, TGF-α, TLR3, WIF-1, and ERBB3, in immunotherapy-treated patients with advanced CC, which were correlated with better prognosis prediction (219). In imiquimod immunotherapy in patients with cervical high-grade squamous intraepithelial lesion (CHSIL), different immune-based cellular biomarkers were identified, including elevated CD4+ T helper cells, M1-polarized macrophages, and DC cells, and achieved favorable predictive performance(AUC 0.95) (220). In clinical trial (NCT04443296, n=27), tumor-infiltrating lymphocytes (TILs) based adoptive cell therapy results in a favorable response rate (75%) in advanced CC associated with low TOX/Foxp3 population and high lymphocyte infiltration (221).

Bioinformatic studies integrated with *in vitro* analysis have also identified candidate biomarkers, including RNA-binding protein signatures with PRPF40B as a potential biomarker for chemotherapy and immunotherapy response (222), immune checkpoint-related biomarkers (Suppressor of TCR signaling 2, Sts-2, co-expressing with multiple checkpoints predicting favorable response to immunotherapy and T cell activity) (223), and immune-related genes (glycolipid transfer protein, GLTP, correlating with CD4+ T cells, macrophages, DCs) (224). These bioinformatics-based immunotherapy response biomarkers need to be validated further to establish their clinical application.

### 2.8 Inflammation-associated metabolic biomarkers

The CC milieu is linked with chronic inflammation and is primarily triggered by a high risk of human papillomavirus infections. The relentless inflammation response transforms the local cellular milieu and rewires the metabolism to encourage cancer survival and progression. The chronic inflammation interrupts the immune response and leads to precancerous progression. Recent studies highlight the specific set of chemokine and receptor genes that serve as critical biomarkers of this inflammatory signaling and immune infiltration, such as CXCL8, CXCL10, CX3CR1, FCGR3B, and SELL, which prolong this persistent inflammatory microenvironment in CC (12). In CC, rapid progression is often linked with significant alterations in metabolism, and HPV infected tissue reprograms this metabolism, which includes the rapid glucose consumption and elevated lactate buildup in local CC tissue, and this shift is triggered by dysregulated expression of some metabolic enzymes like PKM2 and GAD1 (225). The systemic metabolic profile is also altered in CC patients and is not limited to local tissue. This systemic disruption in amino acid, lipid, and carnitine metabolism highlights their potential as non-invasive diagnostic biomarkers (226).

Tracking these metabolic biomarkers offers substantial prognostic value, and specific plasma metabolomic profiles could be used to evaluate a patient’s response and therapeutic effectiveness during Chemotherapy and radiotherapy, yielding promising new targets for both early detection and therapeutic intervention (227).

## 3 Discussion

CC is the fourth most frequent cancer in women in the world. Because of the widespread application of cytological screening programs in the United States, its prevalence and death have been dropping. Yet, there have been geographical differences in CC; members of minority groups, the poor, and those living in rural regions have lower rates of CC immunization, screening, and treatment (228). Reduced CC incidence and death may be accomplished by addressing these gaps through better education, access to care, and extension of screening and vaccination programs (229, 230).

In HPV-positive patients, HPV oncogenes may influence the production of proinflammatory cytokines. Upregulation of these cytokines exacerbates the onset of inflammation after HPV infection. Other variables, such as microRNAs, which are implicated in inflammatory pathways and ageing, cause an increase in proinflammatory cytokines and could be responsible for the amplification of HPV-induced inflammation and CC (231, 232). A vaccine comprising the HPV16 E7 43–77 peptide and the adjuvant unmethylated cytosine-phosphate-guanosine oligodeoxynucleotide evoked strong preventive and therapeutic effects on CC according to cytokine biomarkers. In the vaccine group, the expression of IL-2, IL-12, TNF-α, IFN-γ, CCL-20, CXCL-9, CXCL-10, and CXCL-14 was considerably greater than that in the control group, while the expression of IL-6, IL-10, TGF-β, CCL-2, CCL-3, CCL-5, CXCL-8, MMP-2, MMP-9, and VEGF was significantly lower (233).

While biomarker assays based on nucleic acid changes (DNA methylation patterns, miRNAs) and protein modifications exist, they are rarely used in population screening. Combining nucleic acid and protein-based tests may improve the overall specificity of identifying CIN2+ lesions with a low probability of progressing to CC within the monitoring interval from those with a high risk. The goal is to reduce unnecessary referrals while also reducing the number of false-negative diagnoses. Another roadblock is the development of diagnostic tools for low- and middle-income countries, which is critical for reducing the CC burden (234).

## 4 Future perspective

Biomarkers are crucial in the early detection of diseases and especially cancer, as they can aid in predicting disease progression and therapy response. A single screening of a woman after 35 years of age reduces her chances of dying from cervical cancer by 70%. Regular screening for cervical cancer at 5-year intervals reduces her mortality risk by almost 85% (235). These biomarkers have revealed molecular intricacy that occur during the cervical cancer initiation and progression and could lead to the discovery of new efficient diagnostic tools and therapeutic targets. While biomarker discovery is still in its early stages, however only the conventional biomarkers for CC are being utilized for the diagnosis of clinical cases. There is hope that these potential biomarkers will help in the discovery of new immune cell-based treatments, new therapeutic targets along with early diagnosis and screening of CC which could help in mitigating CC.

Cancer research is going at a greater pace and has revealed a lot of molecular intricacies that could lead to efficient diagnostic tools and therapeutic targets. However, only the conventional biomarkers are still being utilized for the diagnosis and the following points should be taken into consideration in the future research related to CC biomarkers so that these newly discovered biomarkers can be utilized for screening purposes.

- There is a great deal of variability in the biomarkers discovered and analyzed in the preclinical as well as in clinical studies, which means that we need a holistic approach. This holistic approach should consist of an amalgam of omics (genomic, transcriptomic, proteomic studies).
- Personalized medicine has recently made significant progress in becoming a practical clinical tool. Integration of biomarker research would enable the prediction of the efficacy of a given therapy for a certain patient.
- The great deal of variability of biomarkers can be studied through machine learning and computer algorithms to devise a method that is precise and can become a tool for early diagnosis of cervical cancer
- The biomarkers identified in the preclinical level should be studied in clinical studies and could prove beneficial in stratifying sub-types of cervical cancer.

At the initial phases of CC progression, flow cytometry demonstrated a cell phenotype-specific expression profile for VEGFR3, MET, and SLUG, as well as a correlate with the quantity of tumor-infiltrating lymphocytes. These findings, taken combined, add to our understanding of the dynamics that drive angiogenesis and metastasis in HPV-associated CC and may be valuable in the development of new therapeutics (236).

## 5 Conclusion

CC is a life-threatening malignancy, particularly in developing countries, where late-stage diagnosis continues to contribute to high morbidity and mortality among women. Timely detection of CC is crucial for improving overall survival, reducing disease progression, and reducing the likelihood of recurrence. In this review, we discussed in length various biomarkers of an inflammatory nature, including SNPs, DNA methylation, immune cell biomarkers, cytokines, non-coding RNAs, hormones, enzymes, tumor-specific antigens, and cancer cell proliferation markers, highlighting their critical role in detection, prognosis, and therapeutic applications in CC. Various inflammatory cytokines, such as IL-12, IFN-γ, TNF-α, IL-10, and immune cell markers, such as excessive immune cell infiltration, neutrophil-to-lymphocyte ratio, increased B_reg_ cells, and T_reg_ cells, and various other inflammatory molecules have shown their critical role, which could potentially be used as biomarkers for better prognosis, as well as could also be utilized as a therapeutic option for personalized therapy.

In conclusion, we recognize great potential for inflammatory biomarkers and their application in the early screening, diagnosis, and treatment of cervical cancer patients, which could, in turn, improve the quality of life for women.
